# Evaluation of two large language models for intensive care unit discharge decisions: a prospective observational cohort study

**DOI:** 10.1016/j.bjane.2026.844768

**Published:** 2026-05-29

**Authors:** Engin İhsan Turan, Abdurrahman Engin Baydemir, Ebru Kaya, Zehra Polat Turan, Ayça Sultan Şahin

**Affiliations:** a Istanbul Health Science University Kanuni Sultan Süleyman Education and Training Hospital, Department of Anesthesiology, Istanbul, Turkey; b Basaksehir Cam ve Sakura City Hospital, Department of Anesthesiology, Istanbul, Turkey; c Istanbul Health Science University Sisli Hamidiye Etfal Education and Training Hospital, Department of Anesthesiology, Istanbul, Turkey

**Keywords:** Artificial intelligence, Decision making, Decision support systems, Clinical, Intensive care units, Natural language processing, Patient discharge

## Abstract

**Background:**

The aim of this study was to evaluate the effectiveness of two general-purpose Large Language Models (LLMs), ChatGPT and Gemini, in predicting Intensive Care Unit (ICU) discharge decisions (discharge vs. non-discharge). By comparing their outputs with decisions made by ICU physicians, we sought to determine the alignment of AI-generated recommendations with expert clinical judgment and assess their potential as decision-support tools in critical care.

**Methods:**

This prospective observational cohort study was conducted in a tertiary ICU between September 2024 and May 2025. Adult patients (≥ 18 years) requiring ICU discharge decisions were included. Standardized clinical prompts were generated from electronic health records and input into ChatGPT and Gemini. The models’ binary discharge decisions were compared to those of ICU physicians. Model performance was assessed using accuracy, sensitivity, specificity, F1 score, Cohen’s kappa, and McNemar’s test. Discharge was defined as the positive class for all diagnostic performance analyses.

**Results:**

A total of 398 patients were analyzed. ChatGPT demonstrated higher accuracy than Gemini (87.2% vs. 66.3%), with higher sensitivity (85.9% vs. 46.9%) and F1 score (0.890 vs. 0.628), whereas Gemini showed higher specificity (96.2% vs. 89.2%). Agreement with clinician decisions was substantial for ChatGPT (κ = 0.737, p = 0.024) and fair for Gemini (κ = 0.379, p < 0.001). Laboratory markers such as lactate, hemoglobin, and procalcitonin significantly differed between discharged and non-discharged patients.

**Conclusion:**

Large language models may support ICU discharge decisions when guided by structured, guideline-informed prompting. ChatGPT achieved higher overall accuracy, sensitivity, and F1 score, whereas Gemini demonstrated higher specificity.

**Trial registration:**

Externation (Discharge) of ICU, NCT06584890, registered 03 September 2024, prospectively registered, https://register.clinicaltrials.gov/prs/beta/studies/S000EVXZ00000029/recordSummary.

## Introduction

The Intensive Care Unit (ICU) plays a central role in the management of critically ill patients, yet the decision to discharge patients from the ICU remains one of the most complex and high-stake challenges in clinical practice.^1^ Premature discharge may result in readmissions and increased mortality, whereas delayed discharge can lead to inefficient resource utilization, bed shortages, and unnecessary costs.^1^^,^^2^ Clinical guidelines such as those from the Society of Critical Care Medicine (SCCM)^3^ and expert recommendations emphasize the importance of both physiological stability and organizational factors relevant to ICU discharge decisions.^4^ However, the application of these criteria often varies depending on clinician experience and institutional practices, highlighting the need for standardized, objective decision-support mechanisms.

In recent years, Large Language Models (LLMs) like ChatGPT and Gemini have demonstrated promising capabilities in interpreting clinical data and providing recommendations across various domains of medicine.^5^, ^6^, ^7^, ^8^ Despite their general-purpose design, Large Language Models (LLMs) may assist clinicians by synthesizing complex clinical data and providing structured guidance, particularly for decisions like ICU discharge. However, their accuracy and reliability in this context remain insufficiently studied, and real-world integration requires further evaluation. This study aimed to evaluate the effectiveness of two general-purpose AI models, ChatGPT and Gemini, in predicting ICU discharge status (discharge vs. non-discharge) by comparing their outputs with ICU physicians’ decisions, and assessing whether structured, guideline-based prompts improve clinical alignment.

## Methods

### Study design

This was a prospective observational cohort study conducted to evaluate the effectiveness of general-purpose Artificial Intelligence (AI) models, specifically ChatGPT and Gemini, in predicting Intensive Care Unit (ICU) discharge decisions. The study was designed in accordance with the principles of the Declaration of Helsinki. Approval was obtained from the institutional ethics committee prior to data collection (KAEK-2024.08.175), and the study was registered on ClinicalTrials.gov (NCT06584890). Written informed consent was obtained from all patients or their legal representatives before enrollment.

The study was conducted in the Intensive Care Unit (ICU) of a tertiary care hospital. Data collection was performed between September 15, 2024, and May 26, 2025. During this period, all eligible ICU admissions were prospectively screened and evaluated on a daily basis for inclusion in the study. The study was strictly non-interventional; AI-generated outputs were not disclosed to the clinical teams and had no impact on patient management or discharge decisions.

During the preparation of this manuscript, the authors used ChatGPT-4o only to improve language readability. The authors reviewed and edited the content as needed and take full responsibility for the scientific content of the manuscript.

### Participants

All adult patients (aged ≥ 18 years) admitted to the ICU during the study period were screened for eligibility. Inclusion required the availability of sufficient clinical and demographic data in the Electronic Health Records (EHR). Two patient groups were defined: 1) Those discharged from the ICU, for whom data from the day of discharge were used; and 2) Those who remained in the ICU and who died during ICU stay, for whom data from the day prior to death were analyzed. This approach allowed the models to be exposed to both successful and unsuccessful ICU courses.

For patients considered appropriate for ICU discharge, all predefined clinical, physiological, and laboratory variables were systematically recorded from the day of discharge. Likewise, for patients who subsequently died in the ICU, corresponding data were collected in the same standardized manner from the day prior to death. Because data collection was integrated into daily ICU visits and extracted directly from the EHR system, all included patients had complete data for the predefined study variables. ICU readmission was defined as any return to the ICU within 24 hours of ICU discharge and was ascertained using electronic bed-transfer records. Patients were excluded if key clinical data were missing, if they were admitted for palliative or end-of-life care, or if informed consent was not obtained.

### Variables

The primary outcome was ICU discharge status (discharge vs. non-discharge). Model performance was evaluated by comparing AI-generated discharge decisions with physician decisions. The non-discharge group included both patients who remained in the ICU and those who subsequently died, for whom clinical data from the day prior to death were analyzed. The study was designed to evaluate whether contemporary LLM-based systems, when implemented using clinically applicable prompting strategies, can meaningfully align with physician ICU discharge decisions. For all diagnostic performance analyses, discharge was defined as the positive class. Accordingly, sensitivity, specificity, accuracy, and F1 score were calculated based on this definition.

Predictor variables included demographic data (age, gender, comorbidities), clinical indicators (diagnosis, reason for ICU admission), physiological variables (vital signs, Glasgow Coma Scale, ventilator use, inotropic support), laboratory findings (hemoglobin, lactate, creatinine, procalcitonin), and imaging results.

### Subgroup analysis (clinically borderline patients)

A prespecified subgroup analysis was conducted to evaluate model performance in clinically borderline patients, representing cases in which ICU discharge decisions are more challenging and less clinically obvious. Borderline patients were defined as those who were not mechanically ventilated, not intubated, not receiving vasopressor or inotropic support, not on high-flow nasal oxygen therapy, and who were conscious and able to maintain spontaneous respiration.

To avoid inclusion of clinically obvious low-risk cases, patients who were breathing comfortably on room air with a respiratory rate < 20 lt; 20 breaths/min, had stable hemodynamics (mean arterial pressure ≥ 65 mmHg and systolic blood pressure < 180 mmHg), and showed no signs of respiratory distress or neurological impairment were excluded from the borderline subgroup.

### Data sources and measurement

Data were extracted from the hospital’s EHR system and anonymized prior to analysis. All clinical measurements, including laboratory results, vital signs, and physical examination findings, were obtained using standardized hospital protocols to ensure consistency. To evaluate ICU discharge decisions, both models received identical standardized case scenarios derived from the EHR. ChatGPT was accessed through a preconfigured custom GPT that incorporated guideline-based structured instructions and a fixed output framework, such that only the patient scenario was entered for each case. In contrast, Gemini was used in its default interface, and the same standardized prompt text was manually entered together with the patient scenario for each evaluation, without any model-level customization or enforced output structure. These prompts were designed to simulate real-time decision-making and included the following components (Supplementary Material 1, Supplementary Material 2, Supplementary Material 3):

• Demographics (age, sex, comorbidities)
• Reason for ICU admission
• Vital signs (heart rate, blood pressure, respiratory rate, SpO_2_)
• Neurological status (Glasgow Coma Scale)
• Laboratory values (hemoglobin, creatinine, lactate, procalcitonin)
• Mechanical support (mechanical ventilation, inotropic use)
• Physical exam findings (general appearance, respiratory effort, consciousness, edema, signs of hemodynamic instability, abdominal findings).

### GPT model

To evaluate ICU discharge decisions (discharge vs. non-discharge), a custom GPT-based model was developed using structured prompts derived from two key guidelines: the Society of Critical Care Medicine (SCCM) guidelines on ICU admission, discharge, and triage,^3^ and the Plotnikoff et al.^9^ framework on discharge planning and communication. The model was instructed to evaluate patient cases using a standardized five-section output format: 1) Clinical Stability (based on SCCM criteria such as vital signs, GCS, and absence of ICU-specific interventions), 2) Organizational Readiness (per Plotnikoff, focusing on care coordination and communication), 3) Risk Factors for readmission, 4) A binary discharge decision, and 5) A rationale explicitly citing guideline-based justifications. This structured approach enabled consistent, guideline-compliant assessments by the AI.

The GPT-based ICU discharge evaluator used in this study is accessible at the following link: https://chatgpt.com/g/g-682c737d88048191a950d0b7710107af-icu-discharge-evaluator.

Unlike ChatGPT, Gemini does not currently support custom model creation or structured output configuration. Therefore, Gemini 2.5 Flash was used in its default form, responding to standardized clinical prompts without any user-defined formatting or task-specific adaptation.

ChatGPT was accessed via a custom GPT interface based on the GPT-4 architecture, while Gemini 2.5 Flash was used through its default web interface. All model evaluations were conducted using the same institutional network environment. Each patient case was entered and evaluated in a separate session without prior conversational context to prevent memory carryover between cases. No iterative prompting, regeneration, or repeated runs were performed; only a single output per case was used for analysis. Default model parameters were used for both systems, and no manual adjustments to temperature or determinism settings were applied.

### Data governance and privacy

All patient data used in this study were fully de-identified prior to analysis and before being entered into any LLM platform. No directly identifiable information (including names, national identification numbers, hospital numbers, exact dates, or contact details) was used. Only anonymized clinical variables, physiological parameters, and laboratory values were included in structured case templates.

### Bias

To minimize potential bias, clinicians making discharge decisions were blinded to AI predictions. AI models were not updated during the study period to avoid data leakage. Data preprocessing and AI output interpretation were conducted by independent investigators not involved in patient care.

### Power analysis

A priori sample size calculation was performed based on the results of a pilot study involving 50 patients, in which the proportion of discordant pairs between the AI model and physicians was 50%, and the odds ratio was estimated at 1.685. Using G*Power version 3.1.9.4 for a two-tailed McNemar test with a significance level (α) of 0.05 and a desired power (1−β) of 0.95, the required sample size was calculated as 398 patients.

### Statistical analysis

Statistical analyses were performed using IBM SPSS Statistics version 21 (IBM Corp., Armonk, NY). Categorical variables were presented as frequencies and percentages, while continuous variables were summarized as medians with interquartile ranges due to non-normal distributions. Normality of continuous variables was assessed using the Shapiro-Wilk test. Group comparisons for categorical variables were conducted using the Chi-Square test or Fisher’s exact test, as appropriate. The Mann-Whitney *U*-test was used to compare continuous variables between groups.

Agreement between ChatGPT and intensive care physicians was assessed using Cohen’s kappa coefficient. Classification performance metrics, including sensitivity, specificity, accuracy, and F1 score, were calculated based on confusion matrices. Differences between AI model predictions and physician decisions were evaluated using McNemar’s test. To evaluate model performance under more clinically ambiguous conditions, a prespecified subgroup analysis was conducted including patients who were not mechanically ventilated and not receiving vasopressor or inotropic support at the time of evaluation. This subgroup was intended to represent more borderline ICU discharge scenarios. A two-sided p-value of less than 0.05 was considered statistically significant.

## Results

**Figure 1:**
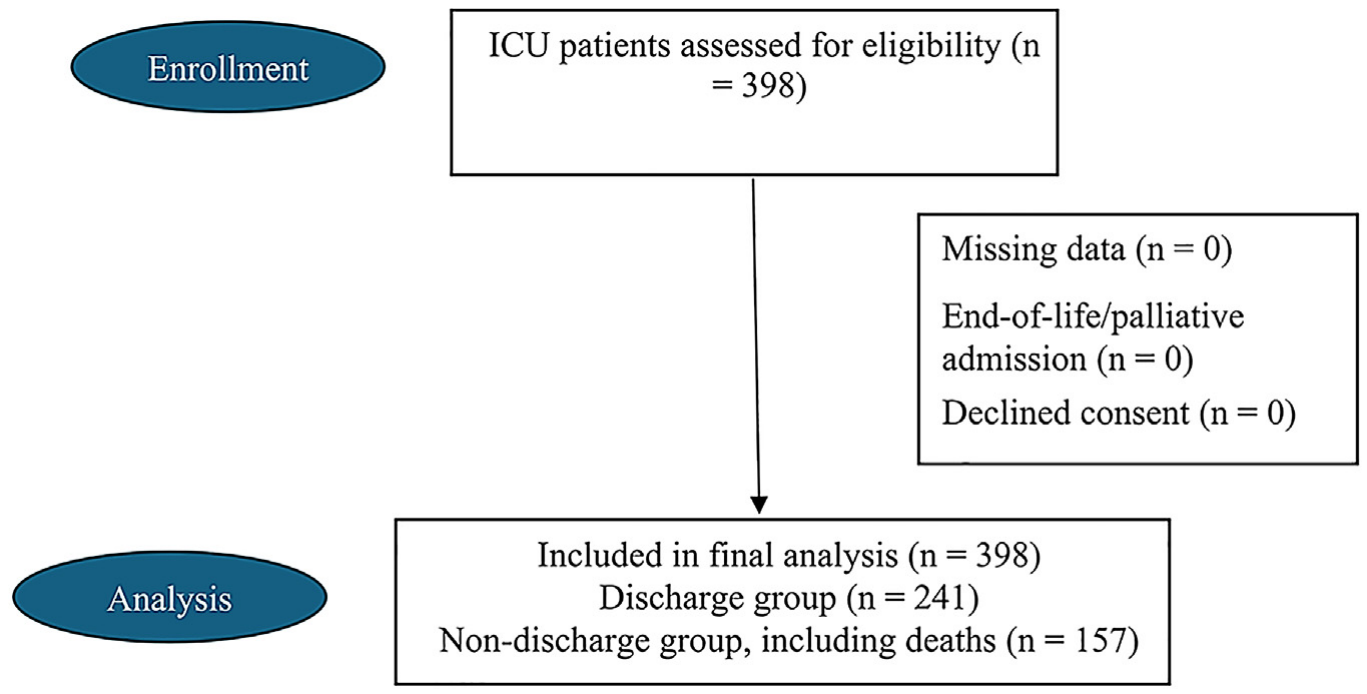
Flowchart of the patients.

A total of 398 adult patients admitted to the ICU were included in the study (Fig. 1). Of these, 147 patients (36.9%) were admitted for postoperative monitoring following major surgical procedures, including abdominal, orthopedic, and neurosurgical interventions. The remaining patients were admitted due to medical conditions such as sepsis, respiratory failure, or cardiac events. All eligible patients were prospectively screened during daily ICU rounds, and all included patients had complete data for the predefined study variables required for the primary analyses. Notably, among all patients included in the study, no ICU readmissions occurred within 24 hours of ICU discharge, supporting the overall safety of the discharge decisions made by clinicians.

Table 1 presents the demographic and clinical characteristics of patients according to their discharge status. The median age was 68 (54–77.5) in the non-discharge group and 65 (52–75) in the discharge group (p = 0.085). Gender distribution was similar between groups, with 83 females and 74 males in the non-discharge group and 137 females and 104 males in the discharge group (p = 0.435). The presence of diabetes mellitus (p = 0.491), coronary artery disease (p = 0.474), heart failure (p = 0.128), stroke (p = 0.444), and COPD (p = 0.790) did not significantly differ between groups. However, the rates of mechanical ventilation and inotropic support were significantly higher in the non-discharge group (p < 0.001 for both).

**Table 1:** Demographic data of the patients.

	Non-Discharge (n = 157)	Discharge (n = 241)	p-value
**Gender (female/male)**	83/74	137/104	p = 0.435^a^
**Age**	68 (54–77.5)	65 (52–75)	0.085
**Diabetes Mellitus (no/yes)**	99/58	159/82	p = 0.491^a^
**Coronary Artery Disease (no/yes)**	129/28	191/50	p = 0.474^a^
**Heart Failure (no/yes)**	129/28	213/27	p = 0.128^a^
**Stroke (no/yes)**	140/17	215/24	p = 0.444^a^
**COPD (no/yes)**	128/29	199/42	p = 0.790^a^
**Mechanical ventilation (no/yes)**	61/96	241/0	p < 0.001^b^
**Inotropic Support (no/yes)**	81/76	240/1	p < 0.001^b^

a Chi-Square test was applied.

b Fisher’s Exact test was applied.

Table 2 compares ChatGPT’s and Gemini’s predictions with the decisions made by intensive care doctors regarding patient discharge. ChatGPT predicted 140 out of 157 non-discharge cases and 207 out of 241 discharge cases correctly. There were 17 false positives and 34 false negatives. The agreement between ChatGPT and physicians was statistically significant (p = 0.024), with a Cohen’s Kappa value of 0.737, indicating substantial agreement. Overall accuracy was 87.2%, sensitivity for predicting discharge was 85.9%, specificity for correctly identifying non-discharge cases was 89.2%, and the F1 score for discharge predictions was 89.0%.

**Table 2:** Comparison of ChatGPT’s and Gemini’s predictions with Intensive care doctor decisions.

		ChatGPT		p^a^	Gemini		p^a^
		Non-Discharge	Discharge		Non-Discharge	Discharge	
**Intensive care doctors**	Non-Discharge	140	17	0.024	151	6	< 0.001
	Discharge	34	207		128	113	

For both models, discharge was treated as the positive class. ChatGPT: accuracy 87.2% (95% CI 83.5–90.2), sensitivity 85.9% (95% CI 80.8–89.9), specificity 89.2% (95% CI 83.3–93.5), F1 score 0.890, κ = 0.737 (95% CI 0.669–0.805), McNemar p = 0.024. Gemini: accuracy 66.3% (95% CI 61.5–70.8), sensitivity 46.9% (95% CI 40.7–53.2), specificity 96.2% (95% CI 91.9–98.4), F1 score 0.628, κ = 0.379 (95% CI 0.310–0.452), McNemar p < 0.001.

a McNemar test was applied.

Among the 398 cases, Gemini correctly identified 151 patients as requiring continued intensive care and 113 patients as appropriate for discharge. The overall accuracy of the model was 0.663. The sensitivity, representing the model’s ability to correctly identify patients to be discharged, was 0.469, while the specificity, indicating correct identification of non-discharge cases, was 0.962. The F1 score, which reflects the balance between precision and recall, was calculated as 0.628. The agreement between Gemini and clinicians, as measured by Cohen’s Kappa, was 0.379.

A prespecified subgroup analysis was performed in clinically borderline patients, defined as those who were not mechanically ventilated and not receiving vasopressor or inotropic support at the time of evaluation. In this subgroup, ChatGPT demonstrated an accuracy of 77.6%, with a sensitivity of 82.4%, specificity of 56.5%, F1-score of 0.857, and fair agreement with clinicians (κ = 0.343). Gemini showed substantially lower overall performance, with an accuracy of 43.2%, sensitivity of 35.3%, specificity of 78.3%, F1-score of 0.504, and slight agreement with clinicians (κ = 0.067) (Table 3).

**Table 3:** Subgroup analysis of borderline patients.

	Accuracy	Sensitivity	Specificity	F1 score	Kappa	95% CI
**ChatGPT**	77.6%	82.4%	56.5%	0.857	0.343	0.208 to 0.482
**Gemini**	43.2%	35.3%	78.3%	0.504	0.067	-0.006 to 0.140

Table 4 presents the comparison of laboratory findings between patients in the non-discharge group and those who were discharged. Median hemoglobin levels were lower in the non-discharge group (8.6 [7.8–10.5]) compared to the discharge group (10.1 [8.9–12.1]) (p < 0.001). Similarly, lactate (3.3 [2.0–10.1] vs. 1.3 [1.0–1.7]), creatinine (1.43 [0.73–2.26] vs. 0.84 [0.64–1.09]), heart rate (90 [83–105.5] vs. 84 [79.5–94]), and procalcitonin levels (1.91 [0.23–6.97] vs. 0.13 [0.06–0.33]) were significantly higher in the non-discharge group, all with p < 0.001. Conversely, SpO_2_ values were slightly lower in the non-discharge group (96 [95–98]) compared to the discharge group (97 [96–98]) (p = 0.048).

**Table 4:** Comparison of laboratory findings of the patients.

	Non-Discharge (n = 157) Median (Q1–Q3)	Discharge (n = 241) Median (Q1–Q3)	p-value^a^
**Hemoglobin**	8.6 (7.8–10.5)	10.1 (8.9–12.1)	< 0.001
**Lactate**	3.3 (2.0–10.1)	1.3 (1.0–1.7)	< 0.001
**Creatinine**	1.43 (0.73–2.26)	0.84 (0.64–1.09)	< 0.001
**Heart Rate**	90 (83–105.5)	84 (79.5–94)	< 0.001
**SpO_2_**	96 (95–98)	97 (96–98)	0.048
**Procalcitonin**	1.91 (0.23–6.97)	0.13 (0.06–0.33)	< 0.001

a Mann-Whitney *U*-test was applied.

## Discussion

In this prospective cohort, both LLM-based systems demonstrated measurable agreement with clinicians in ICU discharge decisions, with the prompt-guided configuration showing higher concordance than the non-customized configuration. However, performance differences were most evident across clinically stable versus unstable presentations, suggesting that structured input and standardized clinical prompting may help LLMs integrate routine ICU variables more consistently. A plausible explanation is that ICU discharge decisions are strongly driven by recognizable patterns of physiological stability and laboratory abnormalities; thus, models may differ in how reliably they extract, prioritize, and weigh these signals when structured prompts and output constraints are applied.

ICU discharge decisions frequently rely on a combination of objective clinical parameters and subjective clinical judgment. Previous work has shown substantial inter-clinician variability in ICU discharge practices, largely attributable to the absence of universally standardized discharge criteria.^10^ Against this background, the relatively weaker alignment observed with the non-customized configuration may reflect not only model limitations but also the inherent inconsistency of the clinical reference standard itself. However, recent efforts to formalize ICU discharge processes are increasingly emphasizing structured, evidence-based frameworks. In this context, the introduction of guideline-informed, standardized prompting in our study was associated with improved alignment between AI outputs and physician decisions, supporting the concept that structured clinical representations may help LLM-based systems capture the implicit heuristics clinicians apply when synthesizing ICU data.^11^ Importantly, during the pilot phase of this study, the same model operating without structured prompts demonstrated substantially lower agreement, suggesting that domain-specific adaptation and clinically grounded prompt design may be at least as critical as the underlying model architecture for effective AI deployment in critical care.

Ethical considerations are central to ICU discharge decision-making, as these decisions not only determine resource utilization but also directly affect patient safety and autonomy. The absence of transparent and standardized discharge policies has been shown to generate ethical tension, inter-clinician conflict, and uncertainty in practice.^12^ From this perspective, structured AI-supported reasoning may offer value not by replacing clinician judgment, but by promoting greater transparency, reproducibility, and ethical consistency in how discharge decisions are formulated.

However, ICU discharge is not determined solely by patient physiology. System-level factors such as protocol gaps, communication failures, and workflow inefficiencies also play a substantial role in discharge decisions and patient safety.^13^ This highlights that AI-based decision support should not be viewed as a standalone solution, but rather as a component of broader, system-oriented strategies aimed at standardizing transitions of care. In this context, our findings align with prior work demonstrating the feasibility of machine learning-based discharge support. For example, Wu et al. reported high agreement between a LightGBM model and physician decisions, with the added benefit of earlier identification of potential discharge candidates.^14^ While their approach relied on structured tabular modeling, our results suggest that LLM-based systems, when clinically guided through structured prompting, may achieve comparable alignment while operating in a more flexible, narrative-compatible format. Previous studies have demonstrated that machine-learning models can support post-ICU risk stratification and discharge-related decision-making.^15^ Together, these observations support the potential role of LLMs as integrative clinical support tools within ethically grounded and system-aware ICU discharge frameworks.

Recent evidence indicates that ICU discharge decisions are influenced by factors extending beyond snapshot physiological measurements. The integration of narrative clinical documentation through natural language processing has been shown to improve discharge modeling and, importantly, to uncover patient subgroups that are poorly captured by conventional structured predictors, including those with psychosocial or surgical complexity.^16^ This suggests that discharge decisions are shaped not only by measurable physiology but also by contextual, organizational, and psychosocial dimensions that are often embedded in daily clinical narratives.

Previous studies have suggested that admission-day variables alone may allow effective early risk stratification for ICU readmission and post-ICU deterioration.^17^ These findings support the concept that clinically meaningful prognostic signals are often present well before discharge and may complement or, in some contexts, outperform discharge-day assessments.

Error pattern analysis revealed distinct behavioral tendencies between the two LLM-based systems. The prompt-guided configuration occasionally favored discharge in cases involving recent seizures or patients receiving high-flow nasal cannula therapy at ≥ 40 L.min^−1^, suggesting a greater tolerance for borderline physiological findings. In contrast, the non-customized configuration demonstrated a predominantly conservative pattern, frequently recommending continued ICU care even in clinically low-risk scenarios, which was reflected in its high specificity but limited sensitivity.

These divergent error profiles indicate that model outputs are shaped not only by the underlying architecture but also by how clinical information is framed and constrained. The conservative bias observed in the non-customized configuration suggests prioritization of isolated physiological abnormalities, whereas the structured prompting strategy appeared to promote a more integrative assessment, yielding a balance between sensitivity and specificity that more closely resembled clinician decision-making. This observation reinforces the concept that prompt design is not merely a technical detail but a central determinant of how LLM-based systems operationalize clinical reasoning.

Importantly, the association between ICU non-discharge and markers such as anemia, hyperlactatemia, renal dysfunction, elevated procalcitonin, mechanical ventilation, and inotropic support holds the biological plausibility of the decision patterns observed. However, while the absence of these features in discharged patients validates their inclusion as model inputs, it also underscores the limitations of single–time-point assessments. The integration of longitudinal parameter trends may allow future AI-supported frameworks to better capture evolving risk profiles and improve performance in more ambiguous discharge scenarios.

### Limitations

This study has several limitations. First, it was conducted in a single tertiary care center, which may limit the diversity of patient populations and care protocols represented. Second, although the AI systems were evaluated using real-time clinical data, they were not embedded within the clinical workflow, and their outputs did not influence actual discharge decisions, thereby limiting assessment of real-world clinical impact. Third, while both systems were tested using structured clinical prompts, the reliance on manually curated input data may not fully reflect the variability, incompleteness, and noise inherent to routine ICU documentation. In addition, model performance may have been influenced by the specific phrasing and formatting of prompts, which were standardized but not externally validated. Moreover, long-term patient-centered outcomes, including post-discharge mortality and readmission beyond the index hospitalization, were not assessed, restricting conclusions regarding downstream safety.

A major methodological limitation is the non-equivalence of AI configurations. ChatGPT was evaluated using a structured, guideline-based custom configuration, whereas Gemini was tested in its default, non-customized form. This design choice reflected the primary aim of determining whether LLM-based systems can meaningfully perform this task under clinically usable conditions rather than comparing intrinsic model capabilities. Consequently, observed differences cannot be attributed to inherent model superiority and may primarily reflect the impact of structured prompting and configuration. A methodologically fair model-to-model comparison would require applying equivalent structured prompts to both systems or evaluating both under default conditions, which should be addressed in future work.

In addition, clinical data were derived from the day of ICU discharge or the day prior to death. Although this reflects real-world decision timing, this approach may have included a substantial proportion of clinically straightforward cases, thereby facilitating discrimination and potentially inflating apparent performance. Importantly, not all patients who subsequently died were uniformly unstable at the time of evaluation, and clinical deterioration occurred after the assessed day in a subset of cases. Future investigations should incorporate longitudinal designs capturing multi-day clinical trajectories, dynamic laboratory trends, and validated severity indices to more rigorously evaluate AI performance under evolving and uncertain discharge conditions.

Finally, although LLMs offer powerful capabilities for clinical reasoning, they remain susceptible to hallucinations, plausible-sounding but incorrect outputs, particularly in the absence of rigorously constrained input structures. While structured prompting likely mitigated this risk, the potential for inaccurate or overconfident responses represents a critical limitation to real-world deployment in safety-critical environments such as intensive care.

### Generalizability

The findings of this study should be interpreted with caution in broader contexts. While the use of structured prompts and standardized criteria provides a foundation for reproducibility, the results may not directly generalize to ICUs with different patient populations, clinical practices, or electronic health record systems. Moreover, the performance of ChatGPT and Gemini may vary across institutions with differing levels of staffing, documentation quality, and discharge policies. Further validation studies in diverse healthcare settings are needed to evaluate whether similar levels of agreement and accuracy can be maintained when these models are integrated into real-time, multidisciplinary ICU decision-making.

## Conclusion

In conclusion, this study supports the feasibility of using general-purpose large language models as decision-support tools for ICU discharge status (discharge vs non-discharge) when implemented with structured, guideline-informed prompting. The prompt-guided configuration demonstrated higher concordance with physician decisions than a non-customized configuration, suggesting that standardization of input structure and task framing may be a key determinant of clinical alignment. Nevertheless, these findings should be interpreted cautiously given the single-center design, the use of a single-time-point assessment, and the non-equivalence of model configurations. Future multicenter studies using standardized, reproducible prompting strategies and longitudinal clinical trajectories are needed to evaluate real-world safety, generalizability, and workflow integration.

## Data availability statement

The data that support the findings of this study are available from the corresponding author upon reasonable request, after de-identification and subject to institutional and ethical approval.

## Compliance with author instructions

We confirm that this manuscript complies with all instructions to authors as outlined by *Brazilian Journal of Anesthesiology*. The study is submitted as an original research article and adheres to the structural and ethical standards required by the journal.

## Authorship compliance

All listed authors meet the authorship criteria defined by the International Committee of Medical Journal Editors (ICMJE). Each author has made substantial contributions to the work, approved the submitted version, and agreed to be accountable for all aspects of the manuscript.

## Ethics approval and consent to participate

This study was approved by the Ethics Committee of the Health Science University Kanuni Sultan Süleyman Training and Research Hospital (Approval Number: KAEK-2024.08.175). Written informed consent was obtained from all participants or their legal surrogates prior to enrollment. The study complies with the principles of the Declaration of Helsinki.

## Use of reporting checklist

This manuscript adheres to the STROBE (Strengthening the Reporting of Observational Studies in Epidemiology) guidelines for observational studies. A completed STROBE checklist is included as a Supplementary File.

## Originality statement

This manuscript has not been published previously and is not under consideration for publication elsewhere. All content is original, and appropriate credit is given for all referenced works. No text or images generated by AI were used in the writing or preparation of the manuscript. ChatGPT 4o was used only to assist in improving the readability of the language.

## Authors’ contributions

Engin İhsan Turan: Study conception and design; AI model development; data interpretation; manuscript drafting, and revision.

Abdurrahman Engin Baydemir: Statistical supervision; methodological refinement, and critical revision.

Ebru Kaya: Patient data collection; clinical interpretation, and coordination of AI model application.

Zehra Polat Turan: Study conception and design; data quality control, and writing.

Ayça Sultan Şahin: Oversight of ICU protocols; ethics documentation, and manuscript revision for intellectual content.

All authors read and approved the final manuscript.

## Funding

The authors did not receive any specific grant from funding agencies in the public, commercial, or not-for-profit sectors for this research.

The authors declare that they have not received any financial support for the conduct of this research or the preparation of this manuscript. Furthermore, there are no conflicts of interest to disclose by any of the authors. This study has not been presented in any congress.

## Declaration of generative AI and AI-assisted technologies in the manuscript preparation process

In this study, ChatGPT 4o has been utilized to enhance the readability of the paper, ensuring that the findings and discussions are accessible and comprehensible to a wider audience.

## Conflicts of interest

The authors declare no conflicts of interest.

## Appendix Supplementary materials

Image, application 1

## Footnotes

Supplementary material associated with this article can be found in the online version at doi:10.1016/j.bjane.2026.844768.
