# Supplementary material for: Evaluation of two large language models for intensive care unit discharge decisions: a prospective observational cohort study
Source: Braz J Anesthesiol. 2026 May 29;76(4):844768. doi: 10.1016/j.bjane.2026.844768 (PMC13312111; doi:10.1016/j.bjane.2026.844768)

**BJAN-D-25-00443_Supplementary Material**

**Supplementary Material 1** Input and output of GPT model.


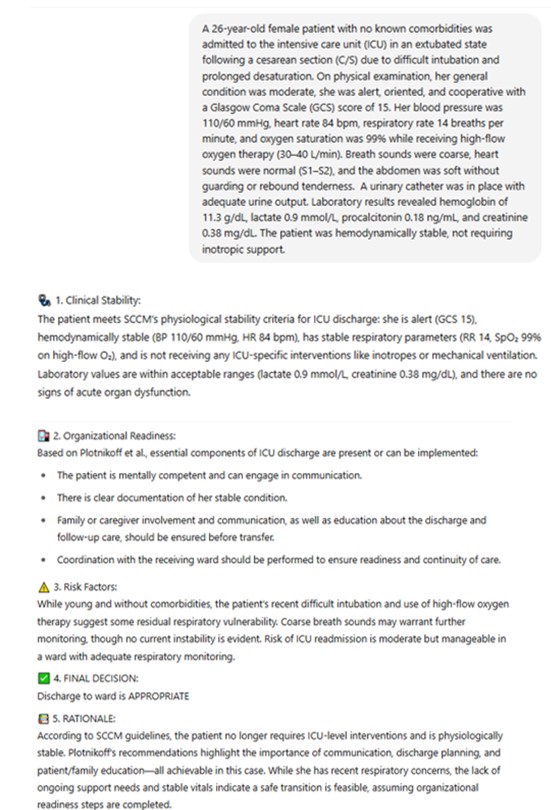


**Supplementary Material 2** Input of Gemini.


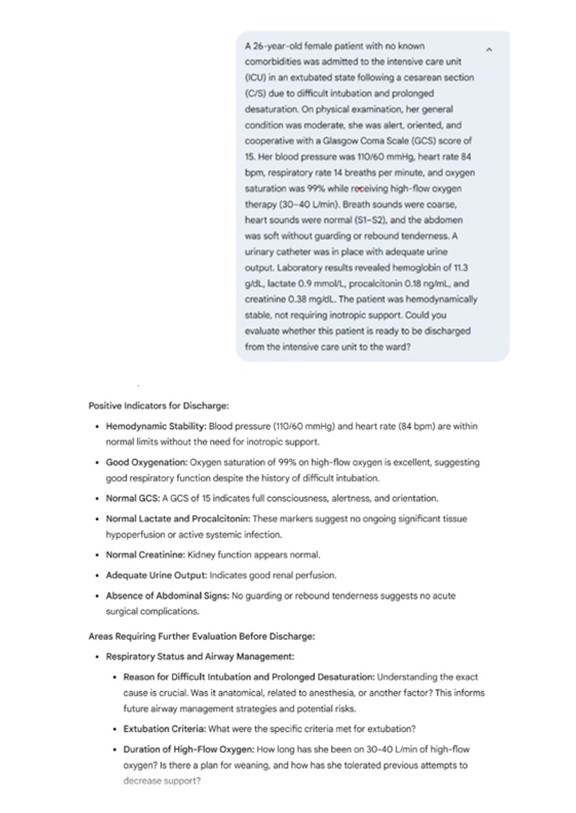


**Supplementary Material 3** Output of Gemini.


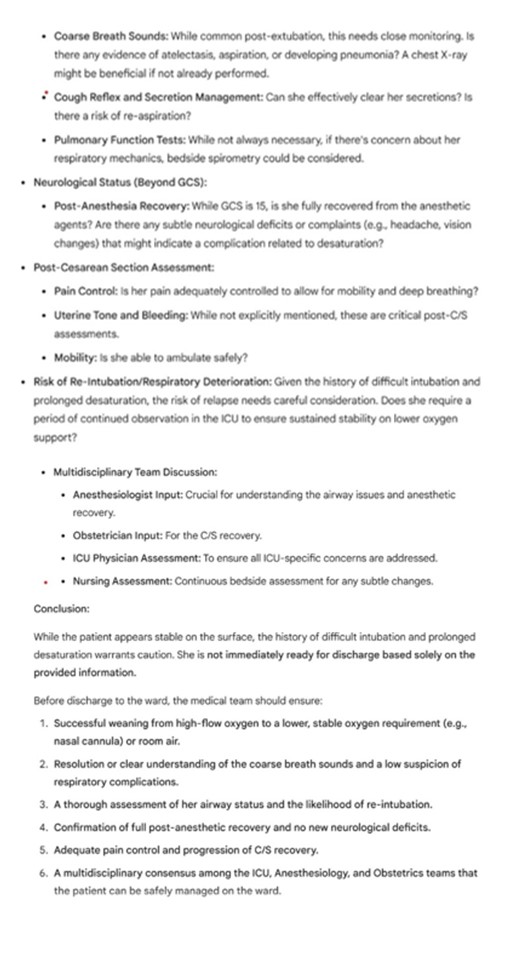

Supplement: Supplementary file 1 [file mmc1.docx]
